# Pelvic exenteration – reconsidering the procedure

**Published:** 2015

**Authors:** N Bacalbasa, I Balescu

**Affiliations:** * “Carol Davila” University of Medicine and Pharmacy, Bucharest, Romania; ** “Ponderas” Hospital, Bucharest, Romania

**Keywords:** advanced pelvic malignancy, pelvic exenteration, postoperative outcome, morbidity

## Abstract

Pelvic exenteration remains one of the most destructive surgical procedures in gynecologic oncology, performed in patients with locally advanced malignancies who were considered for a long time as unresectable. However, for these patients, an aggressive surgical approach seems to be the only potential curative solution. This is a literature review of the most important studies, which analyzes the benefits and the secondary risks of this demanding procedure.

## Introduction

It is well known that pelvic exenteration is one of the most destructive surgical procedures in gynecologic oncology with important secondary effects on the quality of life. However, this is the only potential curative solution for patients with locally invasive cervical cancer, recurrences after cervical cancer, or other gynecologic malignancies such as endometrial cancer, vulvar or ovarian cancer. Initially described by Brunschwig almost 6 decades ago, pelvic exenteration has been performed at that moment with a palliative intent. The first series of pelvic exenteration included 22 cases, reported no intraoperative death but 5 cases encountered severe complications and died during the early postoperative period. Although there was no significant benefit in terms of survival, patients benefited from this aggressive surgery and declared that the quality of life improved when compared with patients who did not undergo surgery [**1**]. Once this statement was approved by the first series of patients submitted by Brunschig to pelvic exenteration, attention was focused on improving intraoperative and postoperative management of these cases in order to decrease the postoperative morbidity and mortality and secondarily to improve the quality of life [**2**]. Although initial rates of morbidity reached up to 40%, these values significantly decreased, being situated nowadays at a mean value of 10%, with a 5-year survival of 40-50% [**1**,**3**-**5**].

**Ontogenetic anatomy**

Pelvic exenteration is in fact the archetype of ultra-radical compartmentalised surgery. The concepts of this radical surgery need to be translated and understood in correlation with the ontogenetic development of the urinary, genital and inferior digestive tract during the embryonic life. According to Hockel, all these 3 compartments have a common origin in the “morphogenetic unit” [**6**]. During the embryonic development, cranial axes come to split this unit in order to recreate the 3 units and their vascular supply, mesenteries and lymphatic drainage. The hindgut is the origin for rectum, mesorectum and anal canal, the Mullerian morphogenetic unit will transform into Fallopian tubes, mesosalpinx, uterine corpus and cervix, mesometrium as well as proximal vagina and mesocolpium while the urogenital sinus and Wolf ducts will give birth to the distal ureters, urinary bladder, urethra and distal vagina. Once these separations are created, they will become natural barriers against tumor propagation in adult. Whenever a tumor appears in any of these compartments, these borders created during the embryonic life will keep them isolated from the other pelvic organs for a long period of time. However, in time, these borders risk to be destroyed by the pelvic neoplasia and local invasion will take place. This seems to be the main mechanism responsible for the apparition of locally advanced pelvic malignancies. These concepts must be well understood in order to perform a radical surgery, which presumes the resection of the invaded organs and their corresponding meso-viscera [**7**]. Moreover, when it comes to rectal resection, all surgeons agree with the necessity of removing the mesorectum too, the same principle being applied in removing the uterine tumours – a mesometrial resection being necessary in all these cases.

According to Hockel’s studies, removing one of these anatomic units en bloc with the afferent viscera is called radical compartmentalized surgery, while removing two or more morphogenetic units represents in fact ultra-radical compartmentalized surgery – pelvic exenteration [**3**].

**The possibility of performing an R0 resection**

Once the notions of anatomy and ultra-radical surgery are well known, R0 resection must be attempted. Nowadays, the only conditions in which R0 resection is not possible are related to sidewall involvement: external iliac vessels, sciatic foramen and obturator nerve or bone invasion. In all the other cases with pelvic confined disease, a complete resection should be tempted. While in cases with locally advanced gynecologic malignancies a radical R0 resection is easier to be obtained, cases presenting recurrent disease being more difficult to be treated by a radical resection. This can be indirectly seen through the per cent of 5-year overall survival in different studies which included recurrent tumours in various proportions (**Table 1**). Also, a higher rate of postoperative complications and even a higher mortality was reported in studies involving resection of pelvic recurrences too [**7**,**14**]. However, the authors consider that resection is perfectly justified even in pelvic recurrences due to the fact that surgery is the only potential chance for an improved survival in these cases [**3**,**7**,**12**,**14**].

**Table 1:** Studies that include recurrent tumours in various proportions

Author and year of the study	No. of cases	Primary tumor	Type of exenteration	R0 resection	Postoperative morbidity (%)	Postoperative mortality (%)	5-year OS
Marnitz, 2006 [**7**]	55	Primary and recurrent cervical cancer	- TE: 51	76%	56,9%	5,5%	36,8%
			- AE: 1				
			- PE: 3				
Berek, 2005 [**8**]	75	Cervical and vaginal cancer: 67	- TE: 46	NR	23%	4,4%	54%
		Uterine cancer: 8	- AE: 23				
			- PE: 6				
Ungar, 2008 [**9**]	41	Cervical cancer	- total SE: 2	NR	22%	0	NR
			- anterior SE: 9				
			- partial anterior SE: 30				
Forner, 2012 [**10**]	27	Vulvar cancer: 27	- TE: 6	74%	66%	0	62%
			- AE: 17				
			- PE: 4				
Forner, 2011 [**11**]	35	Cervical cancer: 35	- TE: 16	97%	50% - minor complications	0	43%
			- AE: 17		22% - major complications		
			- PE: 2				
Hockel, 2006 [**3**]	74	Cervical cancer:11	- LEER: 56	97%	66%	2,7%	56%
		Endometrial Cancer: 2	- TE: 18				
		Vulvar cancer: 5					
Hockel, 2008 [**12**]	102	Cervical cancer: 63	- LEER: 102	97%	70%	2%	55%
		Endometrial Cancer: 8					
		Vulvar cancer: 8					
		Vaginal cancer: 13					
		Other subtypes: 10					
Maggioni, 2009 [**13**]	106	Cervical cancer: 62	- TE: 48	93%	66,6%	0	NR
		Endometrial Cancer: 9	- AE: 53				
		Vulvar cancer: 9	- PE: 6				
		Vaginal cancer: 21					
		Ovarian cancer: 4					
		Uterine leiomyosarcoma: 1					
Schmidt, 2012 [**14**]	282	Cervical cancer (primary advanced or recurrent)	- TE: 119 curative, 143 palliative	65%	51%	5%	64% (curative resection)
			- AE: 11 curative, 3 palliative				19% (palliative resection)
			- PE: 3 curative, 3 palliative				
*SE: supralevator exenteration*							
*TE: total exenteration*							
*AE: anterior exenteration*							
*PE: posterior exenteration*							
*LEER: laterally extended endopelvic resection*							
*NR: not reported*							

Patients presenting tumours fixed to the lower pelvic side wall (up to the level of the sciatic nerve and excluding sciatic foramen) were initially considered as not suitable for radical resection; however, recent studies have demonstrated that they are also candidates for an R0 resection – in all these cases a laterally extended endopelvic resection might be performed. In these cases, resection involves an excision of paravisceral fat pad, obturator internus muscle, iliococcygeus, pubococcygeus, coccygeus muscle and internal iliac vessels system [**12**]. In a study conducted by Hockel et al. on 74 patients with lateral sidewall invasion, an R0 resection using laterally extended exenteration was obtained in 72 cases (97%) [**3**].

Once improved outcomes in terms of survival were obtained, attention was focused on the quality of life of these patients. Roos et al. conducted a study on 32 patients submitted to pelvic exenteration and quality of life was assessed after completing the European Organisation of Research and Treatment of Cancer (EORTC) Core Questionnaire which included five functional scales for physical, emotional, role, cognitive and social functioning. Results were compared to a subgroup of healthy patients and one with cervical cancer; also, a comparison between different subgroups of age, type of surgery and type of tumor were performed. Patients over 60 years had significantly worse scores for physical functioning, dyspnea and sexual activity but a better score for role functioning. Total exenteration had a stronger impact on body image, social functioning and attitude toward the disease when compared to those with partial exenterations. Quality of life was not significantly modified by primary versus recurrent disease. When compared to healthy persons, patients submitted to pelvic exenterations reported similar quality of life [**15**]. The same study reported that women submitted to pelvic exenteration had the same level of emotional functioning as healthy subjects, while cases with recent diagnose of cervical cancer had a lower level of emotional functioning due to the fact that they had no time to adjust to the new situation.

## Conclusions

Although at the beginnings, pelvic exenteration was seen as an extremely destructive procedure associated with unacceptable rates of perioperative morbidity and mortality, in time, this perception has changed; once the operative techniques and postoperative management improved, the early postoperative morbidity and mortality significantly decreased. In the meantime, oncogenic studies of anatomy provided a better understanding of a complete resection of the specimen, en bloc with all the ligaments and meso-viscera, which represent, by themselves, possible routes of microscopic tumoral spread. Once a radical resection is performed and all the meso-viscera are resected, the chances of local recurrence significantly decrease and a better outcome in terms of survival is obtained.
